# Polypus

**Published:** 1831

**Authors:** 


14. *Polypus.*—M. Rigal applied the ligature with success, to a very large polypus, occupying the left nasal fossa, jutting out of the nostril, and pressing behind on the soft palate, causing the tongue to be protruded out of the mouth, and rendering respiration and deglutition difficult. He did not find the hard knot of *Desault*, so useful as it is represented to be, but gave preference to the fillet of *Sauter.*—*Journ. de Chim. Med.*, *April*, 1831.

